# Results from an enhanced surveillance study of laboratory-confirmed acute Lyme disease cases in England between 1 April 2023 and 31 March 2024

**DOI:** 10.1017/S095026882610168X

**Published:** 2026-06-15

**Authors:** Eva Emanuel, Charlotte Robin, Lisa Glaser, Zoë Gibney, Dimple Chudasama, Christina Petridou, Amanda Semper, Rachel Pudney

**Affiliations:** 1 Emerging Infections and Zoonoses, UK Health Security Agency, UK; 2 Rare and Imported Pathogens Laboratory, UK Health Security Agency, UK

**Keywords:** *Borrelia burgdorferi*, Lyme borreliosis, Lyme disease, tick-borne disease, vector-borne disease

## Abstract

In England, Lyme disease (LD) surveillance is based on laboratory-confirmed cases. However, clinical and epidemiological characteristics of these cases are limited; therefore, we conducted an enhanced surveillance study of acute LD in England. Enhanced data were collected through an online questionnaire sent to all laboratory-confirmed acute cases of LD resident in England, with specimen dates between 1 April 2023 and 31 March 2024. The analysis included data from 511/1086 cases. Respondents were representative of age, sex, and region of national LD trends. A total of 57.3% of the respondents reported that they did not realize that they had been bitten by a tick. Among those who remembered a tick bite, 66.1% reported bites close to their home and only 10.6% happened abroad, and 28.5% reported that the tick bite could have occurred in a garden. Most respondents were residents of areas of higher socioeconomic status. Erythema migrans was noted by 70.5% of the respondents. It is important to raise awareness among both patients and clinicians that ticks can be found in urban and suburban parks and gardens, as well as ensuring that wildlife initiatives to increase biodiversity include information on tick prevention and tick checks.

## Background

Lyme disease (LD) is a spirochaetal infection caused by bacteria that belong to the *Borrelia burgdorferi* sensu lato (*B.* *burgdorferi* s.l.) genospecies complex. Transmission to humans and animals occurs through the bite of an infected *Ixodes* spp. tick, most commonly the deer or sheep tick *Ixodes ricinus* in Europe. It is the most common tick-borne infection in humans in the United States and Europe [1] and the most frequently reported vector-borne disease (VBD) in England and Wales [2]. On average, nearly 6% of ticks in England and Wales are infected with Lyme disease bacteria, but this number varies in different areas and between years [3, 4]. The nymphal infection prevalence has been reported to be as high as 30% in site-specific areas in England [3]. Other tick-borne diseases present in the United Kingdom (UK) include tick-borne encephalitis [5], louping ill [6], babesiosis [7], and anaplasmosis [8].

LD most commonly presents as a spreading rash around the bite area known as erythema migrans (EM). EM occurs in approximately 70% of people with LD and typically presents 3–30 days after being bitten by an infected tick and may be associated with flu-like symptoms [9]. Symptoms of early disseminated disease include radiculopathies, cranial nerve palsies, lymphocytic meningitis, and cardiac conduction abnormalities. Late manifestations include Lyme arthritis and neurological complications [9]. There are currently no approved human vaccines available for LD; however, one candidate is in the last stages of clinical development [10].

In England, approximately 1500 laboratory-confirmed cases of LD are reported annually, with an estimated 1000–2000 additional cases each year in England and Wales that are not laboratory-confirmed [2]. In England, the National Institute for Health and Clinical Excellence (NICE) recommends diagnosing and treating patients who present with EM without the need for laboratory testing [9]; therefore, the exact number of cases diagnosed in the community will not be determined from surveillance data. Estimates of LD prevalence from primary care data vary depending on how data are interpreted [11]. Previous studies have highlighted concerns with data quality, including differences in general practitioners’ (GPs) experience and expertise and the availability of different coding options. Additional challenges in estimating the true burden of LD include the following: cases can be asymptomatic; even when symptoms occur, medical attention is not always sought, and therefore, LD is not always medically recognized [12]. Therefore, the current surveillance of laboratory-confirmed LD will demonstrate trends in LD with respect to time, place, and person rather than measuring the incidence or prevalence. LD cases have previously been shown to reflect spatial and temporal variations in tick abundance [4]. The UK Health Security Agency (UKHSA) operates a passive Tick Surveillance Scheme (TSS) which receives tick submissions from members of the public, health practitioners, veterinary practitioners, and wildlife groups [13]. *I. ricinus* submissions to the TSS between 2013 and 2020 had the highest coverage in the south and east of England [13]. LD shows a clear seasonal pattern in England, with the number of reported cases peaking between July and September each year [14] lagging behind the peak period of exposure to ticks in the UK in the spring and summer months [15].

Current LD surveillance in England relies solely on the reporting of laboratory-confirmed cases, with inconsistent recording of patient demographics and risk factors. We conducted an enhanced surveillance system to collect additional information on symptoms, travel, and exposure history, including tick exposure, to better understand the clinical and epidemiological characteristics of laboratory-confirmed cases of acute LD in England.

## Methods

Confirmatory LD testing in England is carried out by the UKHSA Rare and Imported Pathogens Laboratory (RIPL), and data are reported on the Modular Open Laboratory Information System (MOLIS). Data were extracted from MOLIS for laboratory-confirmed cases between 1 April 2023 and 31 March 2024. Enhanced surveillance was conducted through electronic questionnaires sent to all reference laboratory-confirmed acute cases of LD resident in England for the study period.

The following case definitions were used to identify acute cases of LD, as assigned by RIPL on the basis of existing UKHSA LD surveillance criteria:

**Table 1:** Table 1. long description.

A positive Zeus (AccuDiag) Borrelia VlsE1/pepC10 combined IgG/IgM ELISA result on serum confirmed by IgM and IgG immunoblot tests
**OR**
Intrathecal production of specific anti-borrelial antibody consistent with neuroborreliosis
**OR**
A positive *B. burgdorferi* s.l. PCR result
**AND**
A specimen date between 1 April 2023 and 31 March 2024

Individuals classified as past exposures based on serology and IgG responses were excluded from the study to minimize recall bias in reported risk factors and symptoms.

### Data collection

Contact information (phone numbers and email addresses) of eligible patients was collected via the personal demographic service (PDS) [16] from the personal identifiable information available on the laboratory request form. Cases that could not be identified on PDS or lacked valid contact details were excluded from the surveillance. An SMS message or email with a standardized message explaining the rationale of the project and a link to the enhanced surveillance questionnaire was sent to all eligible patients through the government delivery system GovNotify. The questionnaire was completed by the patient or their parent/guardian if the patient was under 16 years of age.

The questionnaire collected information on patient demographics, travel (UK and abroad), outdoor activities, and details of tick bites in the 6 weeks leading up to the patient’s illness and symptoms (Additional file 1).

There was a minimum six-week lag between the laboratory report date and the time at which the questionnaire was sent out to allow the patient’s clinician to notify the patient of their LD diagnosis. To encourage completion of the survey, a note was added to all laboratory reports issued by RIPL to inform clinicians that their patients would receive a questionnaire to complete regarding their LD diagnosis and ask them to inform the patient. At least one week after the first SMS message or email, a second ‘reminder’ message was sent to patients who had not completed the questionnaire. No further reminders were sent, and if patients did not respond, they were classified as non-responders and not contacted again. If a patient was unaware of their diagnosis, they were excluded from the survey, and a clinician subsequently reviewed the case and contacted the patient directly.

### Questionnaire design

The questionnaire was based on previous laboratory specimen questionnaires used by UKHSA and Public Health Scotland for LD surveillance and was reviewed by staff at RIPL and UKHSA’s Medical Entomology and Zoonoses Ecology (MEZE) team (Additional file 1).

### Data analysis

We analysed sociodemographic variables, including age, sex, region, and two UK government metrics – the index of multiple deprivation (IMD) [17] and the rural–urban classification (RUC) [18]. We assessed the representativeness of the respondents to the enhanced surveillance by comparing their sociodemographic characteristics with those of all individuals invited, representing the wider population of acute laboratory-confirmed Lyme disease cases.

The IMD is the official measure of relative deprivation for small areas in England [17] and is commonly used in public health surveillance as a metric to examine social inequalities and their impact on health outcomes, including Lyme disease [20, 21]. The patient postcode was linked to the 2011 lower super output areas (LSOAs), which were then linked to IMD (latest version 2019) to derive an IMD quintile. The RUC is an official statistical classification for England and Wales and is used to distinguish rural and urban areas [18]. The patient postcode was linked to the 2021 LSOA, which was then linked to the 2021 RUC.

The responses to the questionnaire were summarized, and proportions were calculated. The Pearson chi-square test was used to assess the associations between different categorical variables. The Mann–Whitney U test was used to assess whether the median age of those with and without symptoms differed significantly.

Free text fields were manually reviewed, and responses were cleaned and standardized for further analysis.

Data manipulation (cleaning and analysis) was conducted in R v4.3.3 [19] and Excel.

## Results

We identified 1145 eligible individuals who were living in England with laboratory-confirmed serology test results for acute LD undertaken at RIPL, with a sample date between 1 April 2023 and 31 March 2024. Of these, 55 (4.8%) individuals were excluded from the enhanced surveillance: 20 (36.4%) had no valid contact details on PDS, 29 (52.7%) could not be found on PDS, five (9.1%) had not been informed of their Lyme diagnosis at the time of contact, and one (1.8%) was deceased. The remaining 1090 patients were contacted and asked to take part in the enhanced surveillance survey, of whom 515 (47.2%) completed the enhanced surveillance questionnaire. After initial data cleaning, four respondents were excluded from the results: three because their residential postcode was not in England and one because their responses to the questionnaire indicated that they did not have a recent LD infection. Seven respondents completed the enhanced surveillance questionnaire twice, so only their first entry was included in the analysis. The final analysis includes data from 511 cases.

### Demographics

The region with the most people invited to participate in the enhanced surveillance was the South East, with 306 cases, and the region with the fewest people invited was the East Midlands, with 30 cases. The response rate varied by region; the highest response rate was in the South West with 53.3%, and the lowest response rate was in London, at 38.3% (Supplementary materials table 1).

**Figure 1.**
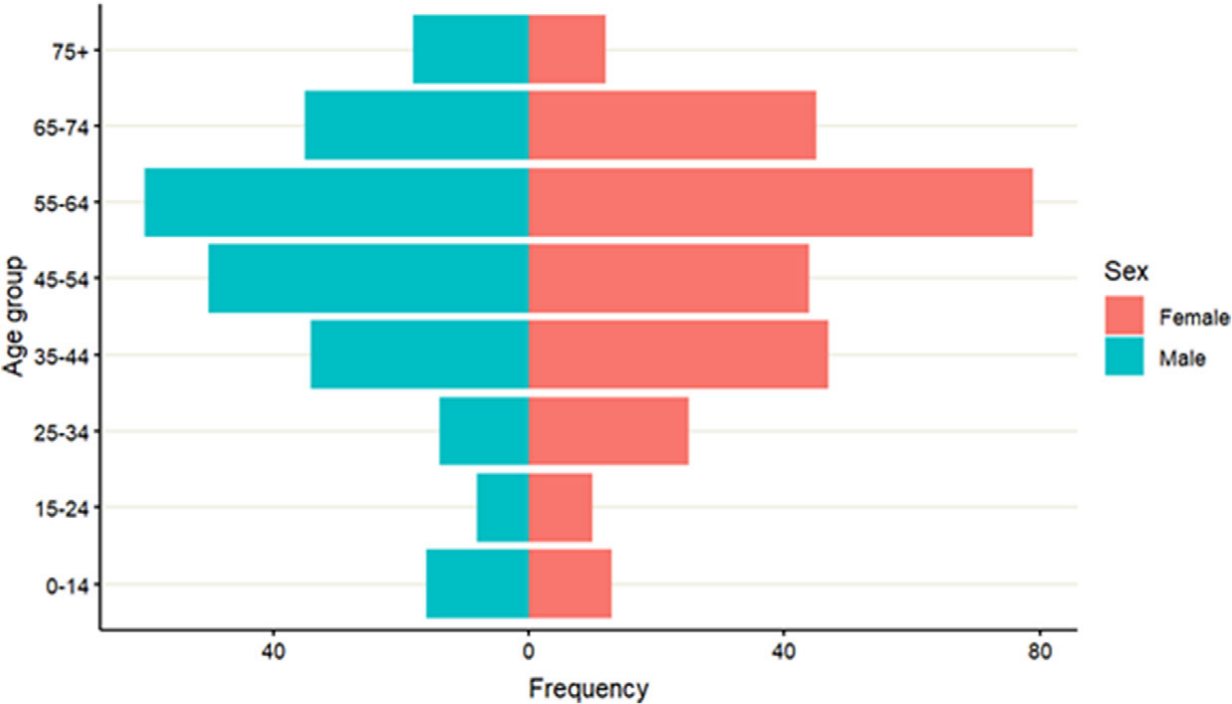
Age–sex pyramid illustrating the age and sex structure of survey respondents with laboratory-confirmed Lyme disease. Figure 1. long description.

For the study period, most of the laboratory-confirmed cases were identified in those aged 35–64 years (56.6%) (Figure 1). The respondents had a median age of 54 years (range: 1–88 years). The response rate varied by age group; the highest response rate was in the 65–74-year group, with 66.1% (80/121), and the lowest response rate was in the 25–34-year group, with 27.7% (39/141) (Supplementary materials table 1).

A similar number of males and females were invited to complete the questionnaire, 48.1% and 50.1%, respectively, and 1.8% had an unknown sex (Figure 1). Of the 511 responses that were included in the analysis, 275 (53.8%) were female, 235 (46.0%) were male, and sex was unknown for 1 (0.2%) respondent. For both males and females, the largest proportion (140/511, 27.4%) of respondents was in the 55–64 age category (M: 59/235, 25.1%; F: 78/275, 28.4%). The group with the fewest respondents (M: 8/235, 3.4%; F: 9/275, 3.3%) was the 15–24-year age group.

Just over half (55.0%) of the respondents lived in the 40% least deprived areas of England. The response rate of individuals in the 80% least deprived quintile was approximately 50%, whereas only 32.1% of respondents in the 20% most deprived quintile took part in the questionnaire.

Most patients invited to participate in enhanced surveillance were residents of urban areas (68.3%, 742/1086), whereas 28.3% (307/1086) were residents of rural areas. Most respondents were from urban areas (64.4%, 329/511), and 35.0% (179/511) of the respondents lived in rural areas. Respondents from rural areas had a higher response rate (58.3%) than those from urban areas (44.3%) or unknown areas.

### Self-reported symptoms

**Figure 2.**
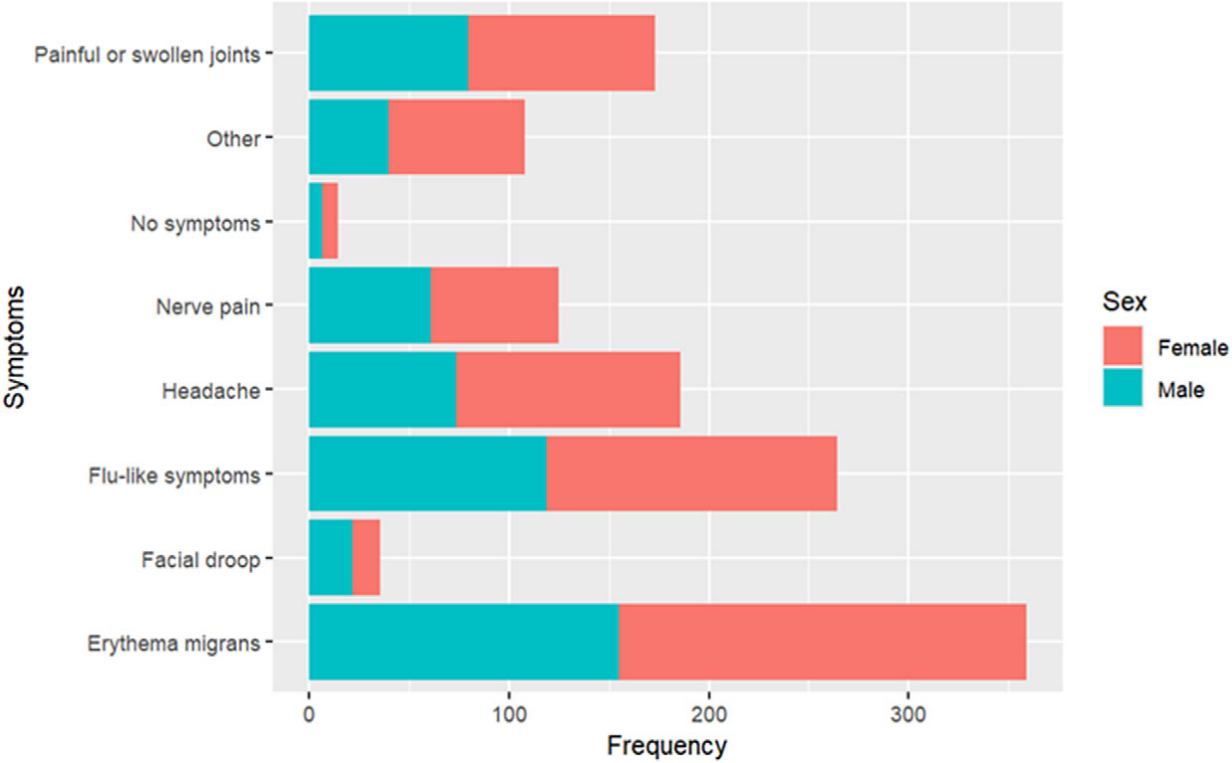
Frequency of reported symptoms among respondents with laboratory-confirmed Lyme disease, stratified by sex. Figure 2. long description.

The most frequently self-reported symptom was ‘spreading red rash, i.e. bull’s-eye or target-shaped rash’, which was reported by 360 (70.5%) respondents (Figure 2) (Supplementary materials Table 2). ‘Flu-like symptoms’, which were defined as three or more symptoms of fever, sweats, chills, tiredness, muscle pain, or joint pain, were reported by 51.7% (264/511) of the respondents. Headache was reported by 36.4% (186/511) of the respondents and was most commonly reported by the 25–34-year age group (53.8%, 21/39). Fifteen respondents (2.9%) reported no symptoms. A total of 108 (21.1%) respondents also reported other symptoms that were described in free text including fatigue (*n* = 27), cognitive dysfunction or brain fog (*n* = 21), and a rash that did not appear to be EM (*n* = 18).

More severe symptoms, such as painful or swollen joints, nerve pain, numbness, and tingling and facial droop, are often consistent with more disseminated LD. Painful or swollen joints were reported by 34.1% (174/511) of respondents and were more common in younger adults, affecting over half of those aged 15–24 years (55.6%, 10/18) and 25–34 years (53.8%, 21/39). Nerve pain, numbness, or tingling was reported by 24.5% (125/511) of the respondents, with the median age of patients reporting this symptom being 53 years (IQR: 41–62 years), and it was most frequently reported by respondents in the 45–54-year age group (35.1%, 33/94). Facial droop was reported by 7.0% (36/511) of the respondents, and the median age of patients reporting facial droop was 44 years (IQR: 20.5–58 years). Facial droop was more frequently reported in patients under 24 years than in those aged 25 years and over (21.3% versus 5.4%; *p* = 0.03). A greater proportion of males under 24 years of age (25%, 6/24) reported facial droop than females (17.4%, 4/23) (*p* = 0.03).

### Tick bites

Fewer than half (42.7%, 218/511) of the respondents recalled being bitten by a tick in the 6 weeks before symptom onset or diagnosis; 234 (45.8%) respondents reported that they could not recall being bitten by a tick, and the remaining 59 (11.5%) were not sure. The 218 respondents who could remember being bitten by a tick were asked further questions on when and where they were bitten.

**Figure 3.**
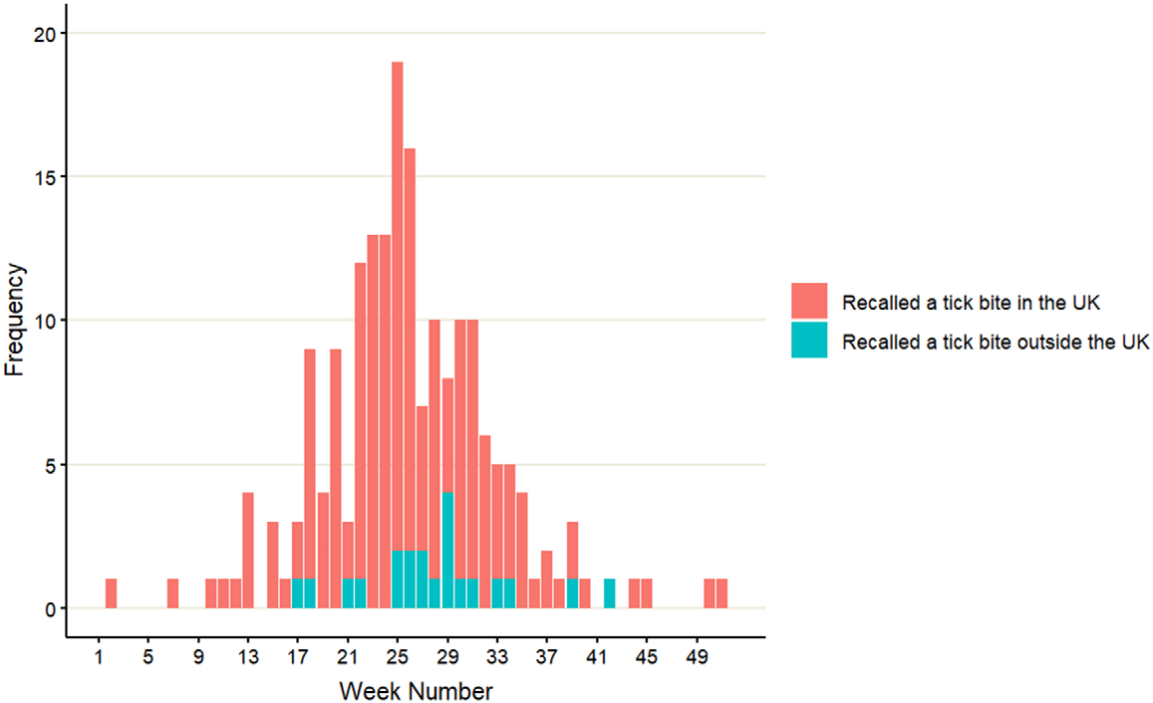
Distribution of recalled tick bites by week number, stratified by whether the bite occurred in the UK or outside the UK (n=218). Week 1 relates to 1 January Figure 3. long description.

Tick bites were most commonly reported in the summer months (Figure 3). The median week number for tick bites that occurred in the United Kingdom was Week 27, which corresponds to July (IQR: Week 18–Week 36) (Figure 3).

The respondents most frequently reported that they were bitten by ticks on their leg or foot (101/218, 46.3%). Thirty-seven respondents reported that they were bitten multiple times on different parts of their body. Younger respondents (<15 years) noted that they were bitten elsewhere on their body, with no respondents under 15 years being bitten on their leg or foot and instead reporting bites on their arms or hands (13.3%, 2/15), head or neck (20%, 3/15), torso (33.3%, 5/15), or multiple bites on different body parts (33.3%, 5/15).

**Figure 4.**
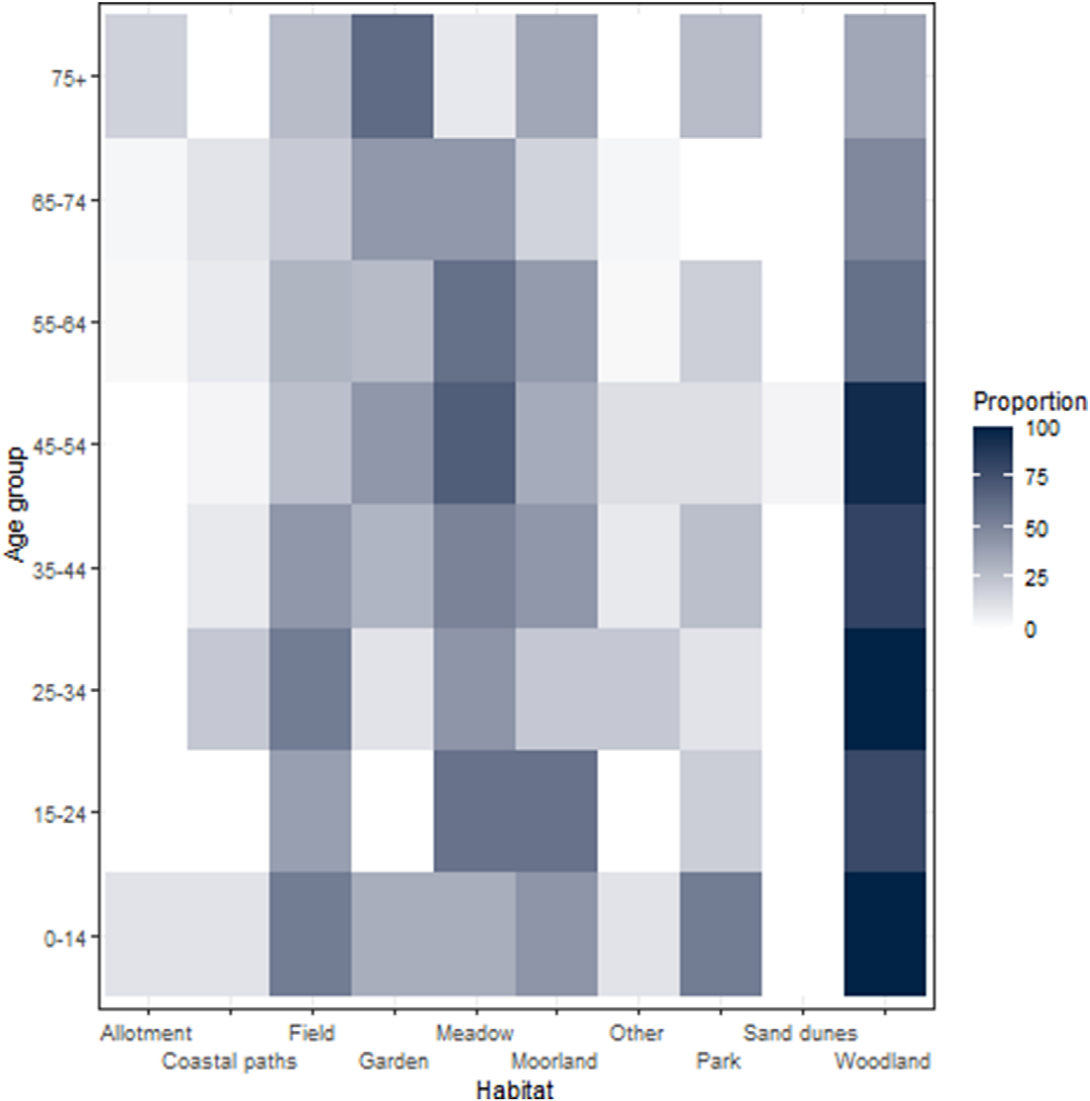
Proportion of respondents reporting the type of environment where a tick bite occurred by age group. Darker shading represents higher proportions, ranging from 0% to 100%. Figure 4. long description.

Nearly half (47.2%) of the respondents reported being bitten by a tick in woodland, and a third (33.5%) reported being bitten by a tick in meadows with long grass. Older respondents (aged over 65 years) more commonly reported being bitten in gardens than those aged under 64 years (40.4% and 22.9%, respectively) (Figure 4) (Supplementary materials table 3).

The majority (66.1%, 144/218) of the respondents reported being bitten by a tick while near home in the United Kingdom, 23.4% reported being bitten by a tick while travelling within the United Kingdom, and 10.6% reported that they were bitten by a tick while outside the United Kingdom.

**Figure 5.**
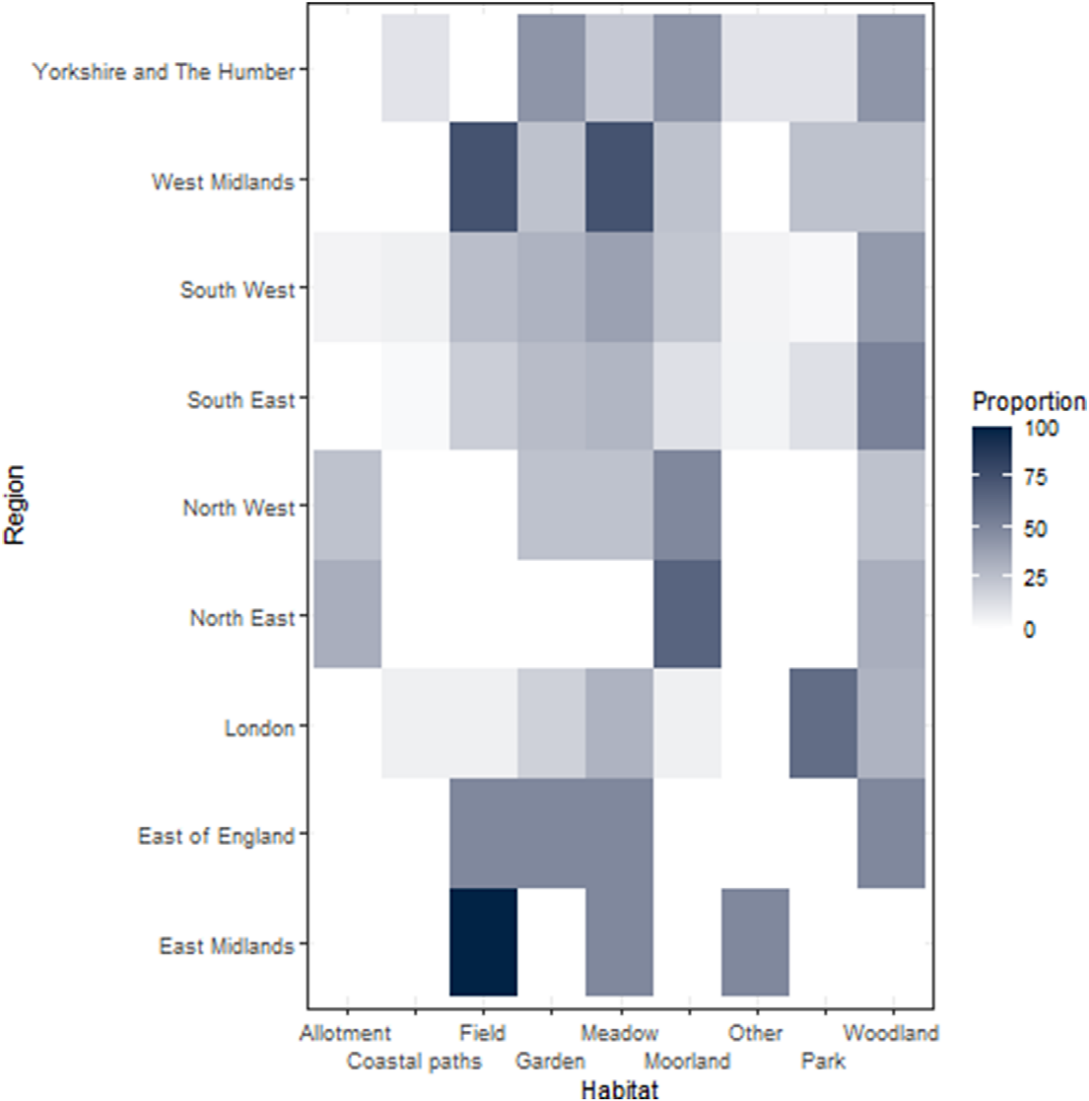
Proportion of respondents reporting the type of environment where a tick bite occurred by region. Darker shading represents higher proportions, ranging from 0% to 100%. Figure 5. long description.

Most respondents who reported being bitten by a tick near home lived in the Southwest (44.4%, 64/144), Southeast (27.8%, 40/144), and London (11.1%, 16/144). The habitats where respondents reported being bitten by a tick varied by region. In the South West, the most common habitats for tick bites were woodland (42.4%, 27/64), meadows with long grass (39.1%, 25/64), and gardens (31.3%, 20/64) (Figure 5) (Supplementary materials Table 4). In the South East, the majority of tick bites occurred in woodland (52.5%, 21/40). In London, the most common habitats were parks and urban green spaces (62.5%, 10/16).

Among those who remembered being bitten by a tick near their home, a slightly greater proportion lived in urban areas (50.7%, 73/144) than in rural areas (48.6%, 70/144), and one respondent had an unknown RUC classification. More people who lived in rural areas (43.0%, 77/179) remembered being bitten by ticks near their home than those who lived in urban areas (22.2%, 73/329). Among the 144 people who reported being bitten by a tick near their home, 41 (28.5%) reported being bitten by a tick in a garden.

### Pets

Overall, 275 (53.8%) respondents reported that they owned pets, of which 252 had pets that were allowed outside the home. Some respondents owned more than one pet; in total, 162 (31.7%) had dogs, 126 (24.7%) had cats, and 26 (5.1%) had other pets. Of the respondents with pets, 73.5% (202/275) reported that they had given their pets regular tick treatments.

### Travel and outdoor activities

In total, 50.9% (260/511) of the respondents reported that they had travelled in the 6 weeks before they noticed symptoms or were diagnosed with LD. A fifth (21.1%, 108/511) reported travelling outside of the United Kingdom in the 6 weeks before they noticed symptoms or were diagnosed with LD. The most frequently reported countries for travel outside the United Kingdom were France (*n* = 15), Sweden (*n* = 13), and the United States (*n* = 10). Of these, 89.8% (97/108) reported taking part in outdoor activities while travelling.

In total, 38.2% (195/511) of the respondents reported travelling within the United Kingdom, of which the majority were within England (71.3%, 139/177). Among those who reported travelling in the United Kingdom, 90.8% (177/195) reported taking part in outdoor activities while travelling. A total of 437 (85.5%) respondents reported taking part in outdoor activities near their home in England. The most common outdoor activities were walking, hiking, gardening, and dog walking.

## Discussion

This study reports findings from the first enhanced surveillance of laboratory-confirmed LD in England. Current surveillance for LD in England is based solely on reporting of laboratory-confirmed cases, with inconsistent recording of patient demographics and risk factors. Our study included data from 511 patients, 47.1% of the total acute LD laboratory-confirmed cases reported to UKHSA RIPL between 1 April 2023 and 31 March 2024.

The age and sex of respondents reflected the overall population affected by LD in England. The majority of acute LD cases were resident in the South West, South East, and London regions of England, which reflects where the majority of laboratory-confirmed LD cases have been reported consistently over the last decade [20, 21] and regions where *I. ricinus* ticks are prevalent [13]. Location of residence does not necessarily reflect where the tick bite occurred, and LD was acquired; overall, 28.2% of respondents reported being bitten by a tick near their home. Local endemicity, clinical awareness of LD, and public health initiatives may influence clinician awareness and patient management [11, 22]. It is therefore possible that where the prevalence is higher, there is greater awareness of LD in these areas, and potentially, more serological testing is requested. Further work is needed to compare the incidence of human cases, the abundance of ticks, and the prevalence of *B. burgdorferi* s.l. in ticks in the same geographic areas.

A fifth of respondents reported recent travel outside the United Kingdom, particularly to France, Sweden, and the United States; however, only 10% or respondents reported a tick bite while travelling outside the United Kingdom. Therefore, this could be an artefact of common travel destinations for people living in the United Kingdom and not that there is more LD in these countries.

In this study, a greater proportion of respondents were residents of the least deprived IMD quintiles. Previous studies in England and Wales [20, 21] and the United States [23, 24] have also reported a greater proportion of LD patients in less deprived areas. This may reflect the type of leisure activities undertaken, available leisure time, and access and attitudes towards the countryside [20]. This could also be due to differences in the awareness of LD, both among clinicians [11, 22] and patients, or differences in health-seeking behaviours. Further qualitative research is needed to examine the reasons for a higher incidence of laboratory-confirmed LD among the least deprived quintiles.

Our data revealed that tick bites occurred year-round, and the seasonality of tick bites aligns with previous publications as well as publications describing *I. ricinus* tick population dynamics in the United Kingdom [8, 25]. Human tick bite records between 2013 and 2018 show that 70% of bites are from *I. ricinus* nymphs [15]; as nymphs are small, they can often remain unnoticed and stay attached for longer [26]. The risk of transmission of *B. Burgdorferi* s.l. to the host increases with the duration of tick feeding [27]; therefore, early recognition and quick removal of ticks are important for reducing the risk of infection. We report that half of the respondents did not recall a tick bite, which may have resulted in delayed tick removal/seeking healthcare advice and treatment. There are existing resources that aim to raise awareness of methods to prevent tick bites and encourage prompt removal of ticks [25]; however, tick identification among members of the public is poor, and there is a lack of knowledge concerning checking for ticks [28]. Continuing to raise awareness of ticks and tick-borne diseases as well as tick bite prevention methods, including the use of insect repellent, regular tick checks, and guidance on safe removal methods, is essential.

In our study, a greater proportion of LD patients lived in rural areas compared to the national population [29]. This aligns with findings from a study in England and Wales [21] and a global meta-analysis [30]. We found that a greater proportion of respondents who were living in rural areas remembered being bitten by a tick near their home compared to those living in urban areas. This could be due to people in rural areas having a greater awareness of ticks and therefore being more likely to check themselves for ticks after outdoor activities.

Among those who remembered being bitten by a tick, the most common habitats where tick bites occurred were woodland and meadows with long grass, which are known to be key habitats of *I. ricinus.* We also found that over a quarter reported that the tick bite could have occurred in a garden. This could be an unintended consequence of wildlife initiatives such as ‘No Mow May’, where individuals are encouraged to let gardens grow to increase wildflower biodiversity for pollinators and, inadvertently, increase the available habitats for ticks [31, 32]. A survey of GPs in the United Kingdom found that fewer than half of England-based respondents recognized that tick habitats may occur in urban and suburban green spaces, as well as rural areas [11]. It is therefore important to raise awareness among both patients and clinicians that ticks can be found in both urban and rural parks and gardens.

*I. ricinus* bites both humans and companion animals and can transmit the Lyme disease pathogen to them [33]. A quarter of the respondents with outdoor pets said that they do not regularly treat their pets with a tick treatment. The use of tick treatments on pets to rapidly kill or repel ticks in addition to performing regular tick checks on pets is recommended [25]. Although the prevalence of LD-causing Borrelia in ticks removed from dogs and cats in the United Kingdom is very low [33], dogs and cats can act as transport hosts for infected ticks, moving them into new locations, including pet owners’ households [33]. It is vital to raise awareness among pet owners of the need to treat pets with tick treatments and check pets for ticks.

LD symptoms can be diverse and nonspecific. In this study, EM was the most commonly reported symptom and was reported by 70.5% of the respondents. The incidence of EM in LD is not clear [34], but a previous study in the United States reported that 70–80% of patients presented with EM [35], whereas in France, 95% of LD patients in primary care presented with EM [36]. In our study, the lower proportion of patients reporting EM was probably due to only recruiting laboratory-confirmed cases, as NICE national guidelines recommend that individuals presenting with EM should be treated empirically with no samples taken for laboratory confirmation [9]. However, it has been reported that over 70% of England-based GPs would access diagnostic testing for a patient with EM, contrary to national guidance [11]. Other countries, including France [37], the Netherlands [38], and Belgium [39], have also reported excessive serological testing of patients with EM seen in general practice. It is important individuals with LD are treated promptly, and unnecessary diagnostic testing can lead to delays in patients receiving treatment or false negatives [9]. Overuse of diagnostic tests could be reduced by improving GPs’ knowledge of LD testing guidelines and symptoms.

We found that a fifth of male and female respondents under 24 years old reported facial droop, which was significantly greater than that of older respondents. Previous studies have shown that 3.5% of patients with Lyme disease develop facial palsy and that younger patients are more likely to develop facial palsy in association with Lyme disease [40, 41]. Other symptoms were also reported by participants including 34.1% reporting painful or swollen joints and 24.5% reporting nerve pain, numbness, or tingling. It is possible that as we only recruited laboratory-confirmed patients, our results were biased towards presentations consistent with more disseminated infections, which required laboratory diagnosis rather than empirical treatment with antibiotics.

One of the main limitations of this enhanced surveillance project was that it did not capture every case of LD that presented to primary care. Laboratory-confirmed cases are an underestimation of the true burden of disease, as they do not include clinically diagnosed cases; estimates from general practice data indicate 1000–2000 cases each year are not laboratory-confirmed [2]. While our methodology of including only acute laboratory-confirmed patients does leave a potential gap in our understanding of LD in England, it would be difficult to conduct enhanced surveillance in primary care outside the remit of a specific research study. Only patients classified as having acute disease by RIPL were included to minimize recall bias. As the questionnaire is completed by the case, responses, particularly for symptoms, can be subjective.

## Conclusion

A large proportion of tick bites were reported to have occurred in the United Kingdom and close to homes, including in gardens, which could be indicative of a shift in habitat and an increased abundance of infected ticks in these areas, or it may be a reflection of human behaviour and tick encounters. It is important to continue enhancing awareness among clinicians and the public that ticks may be present in urban and suburban parks and gardens and ensuring that biodiversity initiatives incorporate information on tick prevention and routine tick checks.

Awareness of early recognition and quick removal of ticks is important for reducing the risk of infection, in addition to reinforcing the need to treat pets with tick treatments and check pets for ticks as additional precautionary measures. Renewed awareness of these guidelines could support greater awareness of the risk of ticks and Lyme disease, particularly with the increased prevalence noted in the United Kingdom.

## Supporting information

10.1017/S095026882610168X.sm001 Emanuel et al. supplementary material Emanuel et al. supplementary material

## Data Availability

The datasets used and/or analysed during the current study are available from the corresponding author on reasonable request.

## List of abbreviations

EM: Erythema migrans
GP: General practitioner
HPT: Health Protection Team
IMD: Index of Multiple Deprivation
LD: Lyme disease
MEZE: Medical Entomology and Zoonoses Ecology
NICE: National Institute for Clinical Excellence
RIPL: Rare and Imported Pathogen Laboratory
RUC: Rural–urban classification
TSS: Tick Surveillance Scheme
UKHSA: UK Health Security Agency
VBD: vector-borne disease

## Supplementary material

The supplementary material for this article can be found at http://doi.org/10.1017/S095026882610168X.

## Patient and public involvement

Patients and/or the public were not involved in the design, or conduct, or reporting, or dissemination plans of this research.

## Long descriptions

### Table 1 Long description

The flowchart consists of three alternatve diagnostic criteria , followed by a final mandatory condition .

Top Section (Alternative Criteria):

- Box 1: A positive Zeus (Accudiag) Borrelia VlsE1/pepC10 combined IgG/IgM ELISA result on serum confirmed by IgM and IgG immunoblot tests

- or

- Box 2: Intrathecal production of specific anti-borrelial antibody consistent with neuroborreliosis.

- or

- Box 3: A positive B. burgdorferi s.l. PCR result

Bottom Section (Mandatory Condition):

-

- Box 4: A specimen date between 1 April 2023 and 31 March 2024.

Navigate back to Table 1.

### [Figure 1] Long description

A population pyramid with a vertical Y axis labeled Age group and a horizontal X axis labeled Frequency. A legend on the right identifies pink bars as Female and teal bars as Male.

From bottom to top, the age groups show the following trends:

* 0 to 14: 16 males and 13 females.

* 15 to 24: 8 males and 10 females.

* 25 to 34: 14 males and 25 females.

* 35 to 44: 34 males and 47 females.

* 45 to 54: 50 males and 44 females.

* 55 to 64: The largest group, with 60 males and 79 females.

* 65 to 74: 35 males and 45 females.

* 75 plus: 18 males and 12 females.

The overall distribution is concentrated in the middle-to-older age brackets, specifically between 35 and 74 years old.

Navigate back to [Figure 1].

### [Figure 2] Long description

The horizontal bar chart plots Symptoms on the Y-axis against Frequency on the X-axis, which ranges from 0 to 400. A legend on the right identifies teal as Male and salmon-pink as Female.

From top to bottom, the symptoms and their approximate frequencies are

* Painful or swollen joints. Male 80, Female 93

* Other. Male 40, Female 68 .

* No symptoms. Male 8, Female 7 .

* Nerve pain. Male 61, Female 64.

* Headache. Male 74, Female 112 .

* Flu-like symptoms. Male 119, Female 145 .

* Facial droop. Male 22, Female 14.

* Erythema migrans. Male 155, Female 204. .

Erythema migrans is the most common symptom

Navigate back to [Figure 2].

### [Figure 3] Long description

The X-axis is labeled Week Number, ranging from 0 to 50, where week 1 relates to the 1 January. The Y-axis is labeled Frequency, ranging from 0 to 15. A legend to the right indicates that salmon-colored bars represent recalling a tick bite in the UK and teal bars represent recalling a tick bite outside the U K.

* The data shows a low frequency of bites from week 0 to 15.

* A significant increase begins around week 17, with multiple peaks.

* The highest frequency of tick bites occurs at week 25.

* Bites recalled outside the UK are represented by small teal segments at the base of the bars, appearing sporadically between weeks 17 and 42, with a small cluster between weeks 25 and 30.

* After week 35, the frequency declines steadily, returning to near-zero levels by week 50.

Navigate back to [Figure 3].

### [Figure 4] Long description

The Y axis represents Age group in years with categories 0-14, 15-24, 25-34, 35-44, 45-54, 55-64, 65-74, and 75 plus. The X axis represents Habitat types where tick bites occurred including Allotment, Coastal paths, Field, Garden, Meadow, Moorland, Other, Park, Sand dunes, and Woodland. A legend on the right titled Proportion uses a blue color gradient where white is 0, light blue is 25 to 50, and dark navy is 75 to 100.

* Woodland: Shows the highest proportion across almost all age groups, appearing as a dark navy vertical band, particularly intense in the 0-14, 25-34, and 45-54 groups.

* Garden: Shows moderate to high proportions, with the darkest cell in the 75 plus age group.

* Meadow, moorland, Parks and Fields: Displays a consistent moderate blue intensity across most age groups.

* Allotment, Coastal paths, Other, and Sand dunes: Generally show light blue to white cells, indicating lower proportions of tick bites occurring in these habitats.

Navigate back to [Figure 4].

### [Figure 5] Long description

The heatmap plots Region on the vertical y axis and Habitat on the horizontal x axis. A color scale legend on the right titled Proportion ranges from 0 in white to 100 in dark blue.

Regions from top to bottom are Yorkshire and The Humber, West Midlands, South West, South East, North West, North East, London, East of England, and East Midlands.

Habitats from left to right are Allotment, Coastal paths, Field, Garden, Meadow, Moorland, Other, Park, and Woodland.

Key data trends include

* The greatest proportion of tick bites in the East Midlands were reported to have occurred in Fields, shown as a dark blue square.

* In the West Midlands the greaterest proportion of tick bites were in shows ffields and meadows.

* In the North East and North West the greatest proportion of tick bites were in moorlands.

* London has its highest proportion of tick bites reported in parks .

Navigate back to [Figure 5].
