# Supplementary material for: Results from an enhanced surveillance study of laboratory-confirmed acute Lyme disease cases in England between 1 April 2023 and 31 March 2024
Source: Epidemiol Infect. 2026 Jun 15;154:e87. doi: 10.1017/S095026882610168X (PMC13312365; doi:10.1017/S095026882610168X)
Supplement: Emanuel et al. supplementary material [file S095026882610168Xsup001.docx]

*Table 1. Sociodemographic characteristics of laboratory-confirmed cases of acute Lyme disease and respondents.*

|  | **Invited** | **Proportion of all invited (%)** | **Responded** | **Proportion of all responses (%)** | **Response rate (%)** |
| --- | --- | --- | --- | --- | --- |
| **Patient region** | | | | | |
| East Midlands | 30 | 2.8 | 15 | 2.9 | 50 |
| East of England | 48 | 4.4 | 22 | 4.3 | 45.8 |
| London | 180 | 16.6 | 69 | 13.5 | 38.3 |
| North East | 35 | 3.2 | 17 | 3.3 | 48.6 |
| North West | 71 | 6.5 | 35 | 6.8 | 49.3 |
| South East | 306 | 28.2 | 154 | 30.1 | 50.3 |
| South West | 274 | 25.2 | 146 | 28.6 | 53.3 |
| West Midlands | 49 | 4.5 | 21 | 4.1 | 42.9 |
| Yorkshire and The Humber | 62 | 5.7 | 32 | 6.3 | 51.6 |
| Unknown | 31 | 2.9 | 0 | 0 | 0 |
| **Age group (years)** | | | | | |
| 0-14 | 90 | 8.3 | 29 | 5.7 | 32.2 |
| 15-24 | 62 | 5.7 | 18 | 3.5 | 29 |
| 25-34 | 141 | 13 | 39 | 7.6 | 27.7 |
| 35-44 | 197 | 18.1 | 81 | 15.9 | 41.1 |
| 45-54 | 183 | 16.9 | 94 | 18.4 | 51.4 |
| 55-64 | 235 | 21.6 | 140 | 27.4 | 59.6 |
| 65-74 | 121 | 11.1 | 80 | 15.7 | 66.1 |
| 75+ | 56 | 5.2 | 30 | 5.9 | 53.6 |
| Unknown | 1 | 0.1 | 0 | 0 | 0 |
| **Sex** | | | | | |
| Female | 544 | 50.1 | 275 | 53.8 | 50.6 |
| Male | 522 | 48.1 | 235 | 46 | 45 |
| Unknown | 20 | 1.8 | 1 | 0.2 | 5 |
| **Index of multiple deprivation quintiles** | | | | | |
| 1 (Most deprived) | 81 | 7.5 | 26 | 5.1 | 32.1 |
| 2 | 157 | 14.5 | 79 | 15.5 | 50.3 |
| 3 | 257 | 23.7 | 125 | 24.5 | 48.6 |
| 4 | 269 | 24.8 | 135 | 26.4 | 50.2 |
| 5 (Least deprived) | 288 | 26.5 | 146 | 28.5 | 50.7 |
| Unknown | 34 | 3.1 | 0 | 0 | 0 |
| **Rural Urban classification** | | | | | |
| Rural | 307 | 28.3 | 179 | 35 | 58.3 |
| Urban | 742 | 68.3 | 329 | 64.4 | 44.3 |
| Unknown | 37 | 3.4 | 3 | 0.6 | 8.1 |
| **Total** | **1086** | **100** | **511** | **100** | **47.1** |

*Table 2. Self-reported symptoms by age and sex (n=511)*.*

|  | **All participants** | | | **Age group** | | | | **Male** | | | **Female** | | | **Unknown sex** | |
| --- | --- | --- | --- | --- | --- | --- | --- | --- | --- | --- | --- | --- | --- | --- | --- |
| **Self-reported Symptoms** | **n** | **%** | **Median age in years (IQR)** | **Aged 64 and under** | **Aged 64 and under (%)** | **Aged 65 and over** | **Aged 65 and over (%)** | **n** | **%** | **median age (years) (IQR)** | **n** | **%** | **Female median age (years) (IQR)** | **n** | **%** |
| Spreading red rash, i.e., bull’s-eye or target-shaped rash | 360 | 70.5 | 55 (42-64) | 281 | 70.1 | 78 | 70.9 | 155 | 67.4 | 54 (42.5-63.5) | 204 | 75.3 | 56 (41-64) | 1 | 10 |
| Flu-like symptoms (3 or more of: fever, seats, chills, tiredness, muscle pain or joint pain) | 264 | 51.7 | 51 (39-62) | 214 | 53.4 | 50 | 45.5 | 119 | 51.7 | 52 (40-61) | 145 | 53.5 | 49 (39-63) | 0 | 0 |
| Headache | 186 | 36.4 | 49 (37.2-60) | 156 | 38.9 | 33 | 30 | 74 | 32.2 | 50 (38.2-59.8) | 112 | 41.3 | 49 (37-60) | 0 | 0 |
| Painful or swollen joints | 174 | 34.1 | 51.5 (39-61.8) | 140 | 34.9 | 33 | 30 | 80 | 34.8 | 51.5 (40-62) | 93 | 34.3 | 50 (36-61) | 1 | 10 |
| Nerve pain/numbness/tingling | 125 | 24.5 | 53 (41-62) | 98 | 24.4 | 27 | 24.5 | 61 | 26.5 | 54 (42-65) | 64 | 23.6 | 50.5 (40.8-60) | 0 | 0 |
| Facial droop | 36 | 7 | 44 (20.5-58) | 31 | 7.7 | 5 | 4.5 | 22 | 9.6 | 45.5 (22-60.2) | 14 | 5.2 | 39.5 (19.2-52.8) | 0 | 0 |
| **Other self-reported symptoms** | | | | | | | | | | | | | | | |
| Fatigue | 27 | 5.3 |  | 22 | 5.5 | 4 | 3.6 | 12 | 5.2 |  | 14 | 5.2 |  | 1 | 10 |
| Cognitive dysfunction/brain fog | 21 | 4.1 |  | 17 | 4.2 | 3 | 2.7 | 6 | 2.6 |  | 14 | 5.2 |  | 1 | 10 |
| Other Rash | 18 | 3.5 |  | 13 | 3.2 | 4 | 3.6 | 6 | 2.6 |  | 11 | 4.1 |  | 1 | 10 |
| Muscle/joint pain | 13 | 2.5 |  | 8 | 2 | 5 | 4.5 | 7 | 3 |  | 6 | 2.2 |  | 0 | 0 |
| Eye issues | 10 | 2 |  | 8 | 2 | 2 | 1.8 | 4 | 1.7 |  | 6 | 2.2 |  | 0 | 0 |
| Heart problems | 9 | 1.8 |  | 9 | 2.2 | 0 | 0 | 6 | 2.6 |  | 3 | 1.1 |  | 0 | 0 |
| Digestive issues | 6 | 1.2 |  | 6 | 1.5 | 0 | 0 | 0 | 0 |  | 6 | 2.2 |  | 0 | 0 |
| Back pain | 5 | 1 |  | 3 | 0.7 | 2 | 1.8 | 2 | 0.9 |  | 3 | 1.1 |  | 0 | 0 |
| Chest pain | 3 | 0.6 |  | 2 | 0.5 | 1 | 0.9 | 1 | 0.4 |  | 2 | 0.7 |  | 0 | 0 |
| Vertigo | 3 | 0.6 |  | 2 | 0.5 | 1 | 0.9 | 0 | 0 |  | 3 | 1.1 |  | 0 | 0 |
| Night sweats | 2 | 0.4 |  | 2 | 0.5 | 0 | 0 | 1 | 0.4 |  | 1 | 0.4 |  | 0 | 0 |
| Swollen joints | 2 | 0.4 |  | 2 | 0.5 | 0 | 0 | 0 | 0 |  | 2 | 0.7 |  | 0 | 0 |
| Tinnitus | 2 | 0.4 |  | 2 | 0.5 | 0 | 0 | 1 | 0.4 |  | 1 | 0.4 |  | 0 | 0 |
| Temporary paralysis | 1 | 0.2 |  | 1 | 0.2 | 0 | 0 | 0 | 0 |  | 1 | 0.4 |  | 0 | 0 |

*Table 3. Habitats where respondents reported being bitten by a tick age group (n=218)*.*

|  | **0-14 years (n=15)** | | **15-24 years (n=7)** | | **25-34 years (n=15)** | | **35-44 years (n=39)** | | **45-54 years (n=38)** | | **55-64 years (n=57)** | | **65-74 years (n=33)** | | **75+ years (n=14)** | | **All (n=218)** | |
| --- | --- | --- | --- | --- | --- | --- | --- | --- | --- | --- | --- | --- | --- | --- | --- | --- | --- | --- |
|  | **n** | **%** | **n** | **%** | **n** | **%** | **n** | **%** | **n** | **%** | **n** | **%** | **n** | **%** | **n** | **%** | **n** | **%** |
| **Allotment** | 1 | 6.7 | 0 | 0 | 0 | 0 | 0 | 0 | 0 | 0 | 1 | 1.8 | 1 | 3 | 2 | 14.3 | 5 | 2.3 |
| **Coastal paths** | 1 | 6.7 | 0 | 0 | 2 | 13.3 | 2 | 5.1 | 1 | 2.6 | 3 | 5.3 | 3 | 9.1 | 0 | 0 | 12 | 5.5 |
| **Field (short grass)** | 5 | 33.3 | 2 | 28.6 | 5 | 33.3 | 10 | 25.6 | 6 | 15.8 | 11 | 19.3 | 6 | 18.2 | 3 | 21.4 | 48 | 22 |
| **Garden** | 3 | 20 | 0 | 0 | 1 | 6.7 | 7 | 17.9 | 10 | 26.3 | 10 | 17.5 | 12 | 36.4 | 7 | 50 | 50 | 22.9 |
| **Meadow (long grass)** | 3 | 20 | 3 | 42.9 | 4 | 26.7 | 12 | 30.8 | 16 | 42.1 | 22 | 38.6 | 12 | 36.4 | 1 | 7.1 | 73 | 33.5 |
| **Moorland/upland/heathland** | 4 | 26.7 | 3 | 42.9 | 2 | 13.3 | 10 | 25.6 | 8 | 21.1 | 15 | 26.3 | 5 | 15.2 | 4 | 28.6 | 51 | 23.4 |
| **Other** | 1 | 6.7 | 0 | 0 | 2 | 13.3 | 2 | 5.1 | 3 | 7.9 | 1 | 1.8 | 1 | 3 | 0 | 0 | 10 | 4.6 |
| **Park/urban green space** | 5 | 33.3 | 1 | 14.3 | 1 | 6.7 | 6 | 15.4 | 3 | 7.9 | 7 | 12.3 | 0 | 0 | 3 | 21.4 | 26 | 11.9 |
| **Sand dunes** | 0 | 0 | 0 | 0 | 0 | 0 | 0 | 0 | 1 | 2.6 | 0 | 0 | 0 | 0 | 0 | 0 | 1 | 0.5 |
| **Woodland** | 9 | 60 | 4 | 57.1 | 9 | 60 | 19 | 48.7 | 22 | 57.9 | 22 | 38.6 | 14 | 42.4 | 4 | 28.6 | 103 | 47.2 |

**Respondents reported more than one habitat.*

*Table 4. Habitats where respondents reported being bitten by ticks if the bite occurred near home by region (n=144)*.*

|  | **East Midlands (n=2)** | | **East of England (n=2)** | | **London (n=16)** | | **North East (n=3)** | | **North West (n=4)** | | **South East (n=40)** | | **South West (n=64)** | | **West Midlands (n=4)** | | **Yorkshire and The Humber (n=9)** | | **All (n=144)** | |
| --- | --- | --- | --- | --- | --- | --- | --- | --- | --- | --- | --- | --- | --- | --- | --- | --- | --- | --- | --- | --- |
|  | **n** | **%** | **n** | **%** | **n** | **%** | **n** | **%** | **n** | **%** | **n** | **%** | **n** | **%** | **n** | **%** | **n** | **%** | **n** | **%** |
| **Allotment** | 0 | 0 | 0 | 0 | 0 | 0 | 1 | 33.3 | 1 | 25 | 0 | 0 | 3 | 4.7 | 0 | 0 | 0 | 0 | 5 | 3.5 |
| **Coastal paths** | 0 | 0 | 0 | 0 | 1 | 6.3 | 0 | 0 | 0 | 0 | 1 | 2.5 | 4 | 6.3 | 0 | 0 | 1 | 11.1 | 7 | 4.9 |
| **Field (short grass)** | 2 | 100 | 1 | 50 | 1 | 6.3 | 0 | 0 | 0 | 0 | 8 | 20 | 17 | 26.6 | 3 | 75 | 0 | 0 | 32 | 22.2 |
| **Garden** | 0 | 0 | 1 | 50 | 3 | 18.8 | 0 | 0 | 1 | 25 | 11 | 27.5 | 20 | 31.3 | 1 | 25 | 4 | 44.4 | 41 | 28.5 |
| **Meadow (long grass)** | 1 | 50 | 1 | 50 | 5 | 31.3 | 0 | 0 | 1 | 25 | 12 | 30 | 25 | 39.1 | 3 | 75 | 2 | 22.2 | 50 | 34.7 |
| **Moorland/upland/heathland** | 0 | 0 | 0 | 0 | 1 | 6.3 | 2 | 66.7 | 2 | 50 | 5 | 12.5 | 15 | 23.4 | 1 | 25 | 4 | 44.4 | 30 | 20.8 |
| **Other** | 1 | 50 | 0 | 0 | 0 | 0 | 0 | 0 | 0 | 0 | 2 | 5 | 3 | 4.7 | 0 | 0 | 1 | 11.1 | 7 | 4.9 |
| **Park/urban green space** | 0 | 0 | 0 | 0 | 10 | 62.5 | 0 | 0 | 0 | 0 | 5 | 12.5 | 2 | 3.1 | 1 | 25 | 1 | 11.1 | 19 | 13.2 |
| **Woodland** | 0 | 0 | 1 | 50 | 5 | 31.3 | 1 | 33.3 | 1 | 25 | 21 | 52.5 | 27 | 42.2 | 1 | 25 | 4 | 44.4 | 61 | 42.4 |

**Respondents reported more than one habitat.*
